# Evolution of the coronavirus spike protein in the full-length genome and defective viral genome under diverse selection pressures

**DOI:** 10.1099/jgv.0.001920

**Published:** 2023-11-24

**Authors:** Ching-Hung Lin, Hon-Man-Herman Tam, Cheng-Yao Yang, Feng-Cheng Hsieh, Jiun-Long Wang, Chun-Chun Yang, Hsuan-Wei Hsu, Hao-Ping Liu, Hung-Yi Wu

**Affiliations:** ^1^​ Graduate Institute of Veterinary Pathobiology, College of Veterinary Medicine, National Chung Hsing University, Taichung 40227, Taiwan, ROC; ^2^​ Department of Veterinary Medicine, College of Veterinary Medicine, National Chung Hsing University, Taichung 40227, Taiwan, ROC; ^3^​ Division of Chest Medicine, Department of Internal Medicine, Taichung Veterans General Hospital, Taichung 40705, Taiwan, ROC; ^4^​ Department of Post-Baccalaureate Medicine, College of Medicine, National Chung Hsing University, Taichung 40227, Taiwan, ROC; ^5^​ Department of Life Sciences, National Chung Hsing University, Taichung 40227, Taiwan, ROC

**Keywords:** coronavirus, defective viral genome, evolution, selection pressure, spike protein

## Abstract

How coronaviruses evolve by altering the structures of their full-length genome and defective viral genome (DVG) under dynamic selection pressures has not been studied. In this study, we aimed to experimentally identify the dynamic evolutionary patterns of the S protein sequence in the full-length genome and DVG under diverse selection pressures, including persistence, innate immunity and antiviral drugs. The evolutionary features of the S protein sequence in the full-length genome and in the DVG under diverse selection pressures are as follows: (i) the number of nucleotide (nt) mutations does not necessarily increase with the number of selection pressures; (ii) certain types of selection pressure(s) can lead to specific nt mutations; (iii) the mutated nt sequence can be reverted to the wild-type nt sequence under the certain type of selection pressure(s); (iv) the DVG can also undergo mutations and evolve independently of the full-length genome; and (v) DVG species are regulated during evolution under diverse selection pressures. The various evolutionary patterns of the S protein sequence in the full-length genome and DVG identified in this study may contribute to coronaviral fitness under diverse selection pressures.

## Impact Statement

In this study, we experimentally explored how a coronavirus reacted to diverse selection pressures in terms of alterations in the S protein sequence in the full-length genome and the defective viral genome (DVG). Based on the results, it is suggested for the first time that DVG species can be regulated, undergo mutations, evolve independently and exhibit the potential to recombine with the full-length genome under diverse selection pressures. Thus, in addition to the diverse evolutionary patterns identified in the full-length genome, the aforementioned evolutionary features of the DVG may also be the strategies by which coronaviruses can adapt to different environments for viral fitness. The identified evolutionary patterns are expected to provide information to explain the possible evolutionary trends during the coronavirus pandemic, contributing to disease control and public health.

## Introduction

Coronaviruses (CoVs) are critical human pathogens that cause widespread and costly diseases, including severe acute respiratory syndrome (SARS) [1], Middle East respiratory syndrome (MERS) [2] and a novel viral pneumonia (officially called COVID-19) [3, 4]. CoVs in animals including porcine epidemic diarrhoea virus (PEDV), infectious bronchitis virus (IBV) and bovine coronavirus (BCoV), have also led to economic losses worldwide [5].

CoVs have a single-stranded, positive-sense RNA genome which is 26–30 kb in length [6, 7]. The nonstructural proteins are encoded by the genome, and four major structural proteins, the spike (S), envelope (E), membrane (M) and nucleocapsid (N) proteins, are encoded by subgenomic mRNAs (sgmRNAs) [8]. Among the structural proteins, the S protein is involved in receptor recognition, viral attachment and entry into host cells. The S protein mainly consists of the S1 subunit, which is responsible for receptor binding, and the S2 subunit, which functions in membrane fusion [9]. The S protein gene (~4 kb) can undergo mutations frequently and thus can adapt to changed infection environments and to circumvent selection pressure, such as immunity conferred by natural infection or vaccination [10, 11].

In addition to the genome and sgmRNAs, the CoV defective viral genome (DVG) has been suggested to be abundantly synthesized during natural infection in cell cultures [12]. The DVG is a truncated version of the CoV full-length genome, presumably synthesized by a copy-choice template-switching mechanism. DVGs in other RNA viruses have been indicated to be associated with virus replication [13] and pathogenicity [14]; however, the role of the CoV DVG and its evolutionary biology remain unclear.

After natural infection, CoVs, including SARS-CoV-2, can also establish persistence [15–20]. To establish persistence, the CoV may modify its biological characteristics, including alteration of genome structures, to react to environmental changes [21]. Similarly, host cells can resist CoV infection by adjusting their physiological condition. Consequently, a coevolution process between the virus and host cells is established [22]. In addition, the host can also launch an immune response to counteract viral infection. It has been shown that influenza A virus, hepatitis C virus and poliovirus can modify their genetic composition to escape adaptive immunity [23–25]. On the other hand, during a pandemic such as COVID-19, antivirals such as remdesivir (GS-5734) have been frequently used to shorten recovery time in adults with COVID-19 [26]. It has been demonstrated that, under the selection pressure of antiviral drugs, human immunodeficiency virus-1 (HIV-1), hepatitis C virus, influenza A virus and coronavirus can evolve to develop drug resistance by alterations of genome structure [27–32]. Consequently, the selected virus with drug resistance may repopulate within hosts or transmit to others, posing a public health concern.

Due to a lack of proofreading capability, RNA viruses including CoVs can diversify easily into different populations and thereby evolve with time under constant selection pressures for viral fitness [33]. This feature thus results in a diverse population of viruses with altered genome structures during replication. Taking advantage of this feature, RNA viruses may weather selection pressures for survival. However, how CoVs evolve by altering their genome structures under multiple selection pressures such as persistence, immune responses and antiviral treatment is of interest, but this process remains unknown. Furthermore, in addition to the full-length genome, the evolutionary biology of the CoV DVG under multiple selection pressures has not been previously studied. In this study using BCoV as a test model, we sought to identify the dynamic evolutionary patterns of the S protein sequence in the full-length genome and DVG under diverse selection pressures. The results are expected to provide information that may contribute to explaining the evolutionary trends of CoV during the pandemic and thus are important in public health.

## Methods

### Virus and cells

The Mebus strain of BCoV (GenBank: U00735.2), which was obtained from David A. Brian (University of Tennessee, TN, USA), and human rectal tumour (HRT)-18G cells (ATCC, CRL-11663) were used for the study. BCoV was grown in HRT-18G cells. HRT-18G cells were grown in Dulbecco’s modified Eagle’s medium (DMEM) supplemented with 10 % FBS (HyClone) at 37 °C with 5 % CO_2_ as previously described.

### Establishment of a single, double or triple selection pressure(s)

To establish persistence, HRT-18G cells were infected with BCoV at an m.o.i. of 1. The infected cells were then passaged every 5 days. The supernatant and cellular RNA were collected at each passage. The viral and cellular RNA collected at 50 and 100 days of persistent infection were designated P50d and P100d, respectively.

For the double selection pressures of persistence and innate immunity induced by polyinosinic:polycytidylic acid (poly IC), persistently infected HRT-18G cells at 50 days were treated with poly IC at a final concentration of 1 µg ml^−1^. This stage of infection was defined as virus passage 0 (VP0). After 5 days of treatment, the HRT-18G cells were passaged (VP1) and then treated with poly IC at a final concentration of 1 µg ml^−1^. The step of virus passage followed by poly IC treatment was repeated until VP10. The supernatant and cellular RNA were collected at each passage. The viral and cellular RNA collected at VP10 was designated P100dIC.

For the double selection pressures of persistence and antiviral activity, persistently infected HRT-18G cells at 50 days were treated with antiviral remdesivir (GS-5734) at a final concentration of 10 µM. This stage of infection was defined as virus passage 1 (VP0). After 5 days of treatment, the HRT-18G cells were passaged (VP1) and then treated with the antiviral GS-5734 at a final concentration of 10 µM. The step of virus passage followed by treatment with GS-5734 was repeated until VP10. The supernatant and cellular RNA were collected at each passage. The viral and cellular RNA collected at VP10 was designated P100dGS.

For the triple selection pressures of persistence, innate immunity and antiviral activity, persistently infected HRT-18G cells at 50 days were treated with poly IC and GS-5734 at final concentrations of 1 µg ml^−1^ and 10 µM, respectively. This stage of infection was defined as virus passage 1 (VP0). After 5 days of treatment, the HRT-18G cells were passaged (VP1) and then treated with poly IC and GS-5734 at final concentrations of 1 µg ml^−1^ and 10 µM, respectively. The step of virus passage followed by treatment with poly IC and GS-5734 was repeated until VP10. The supernatant and cellular RNA were collected at each passage. The viral and cellular RNA collected at VP10 was designated P100dICGS.

### Determination of genome structure by RT-PCR and sequencing

Cellular RNA was extracted with TRIzol (Thermo Fisher Scientific) according to the manufacturer’s protocol. A total of 1500 ng of extracted RNA was used for cDNA synthesis. For this, the extracted RNA was mixed with 2 µM primer BCVEND2(+) (Table S1, available in the online version of this article), denatured at 95 °C for 5 min and placed on ice for 1 min. One microlitre of SuperScrip III reverse transcriptase (RT; 200 U µl^–1^) (Thermo Fisher Scientific) was then added to the mixture, followed by incubation at 42 °C for 50 min and 70 °C for 15 min. For determination of the S protein gene sequence, multiple sets of primers (Table S1) were used to amplify the S protein gene fragments using AccuPrime Taq DNA Polymerase (Thermo Fisher Scientific) followed by sequencing. For determination of the sequence of DVG species, various primer sets (Table S1) located at the 5′ terminus of the genome and S protein gene were selected for PCR followed by sequencing. Amplification was performed under the following conditions: 94 °C for 5 min, 94 °C for 1 min, 55 °C for 1 min and 72 °C for 2 min for 35 cycles followed by a single final extension step (72 °C, 5 min). Triplicate PCR products were amplified and sequenced.

### RT-qPCR

To determine the replication efficiency of the selected virus variants, WT, P50d, P100d, P100dIC, P100dGS and P100dICGS were used for infection of fresh HRT-18 cells. At 2, 24 and 48 h post-infection, total cellular RNA was collected. Ten micrograms of TRIzol-extracted total cellular RNA was used for the RT reaction. For quantitative (q)PCR, SYBR green amplification mix (Roche Applied Science) and oligonucleotides (Table S2) binding to the 1a/1b protein-encoding gene and N protein-encoding gene were used according to the manufacturer’s protocol. The data were normalized to the levels of 18S rRNA. The reactions were performed with an initial preincubation at 95 °C for 5 min followed by 35 amplification cycles of 95 °C for 15 s and 60 °C for 30 s.

### Sequence analysis

Sequences were edited using the DNASTAR Lasergene v.6 SeqMan II and EditSeq programs. Nucleotide sequences were aligned by using the DNASTAR Lasergene v.6 MegAlign program.

## Results

### Different evolutionary patterns were observed in S protein sequences during single, double and triple selection pressure(s)

**Fig. 1.**
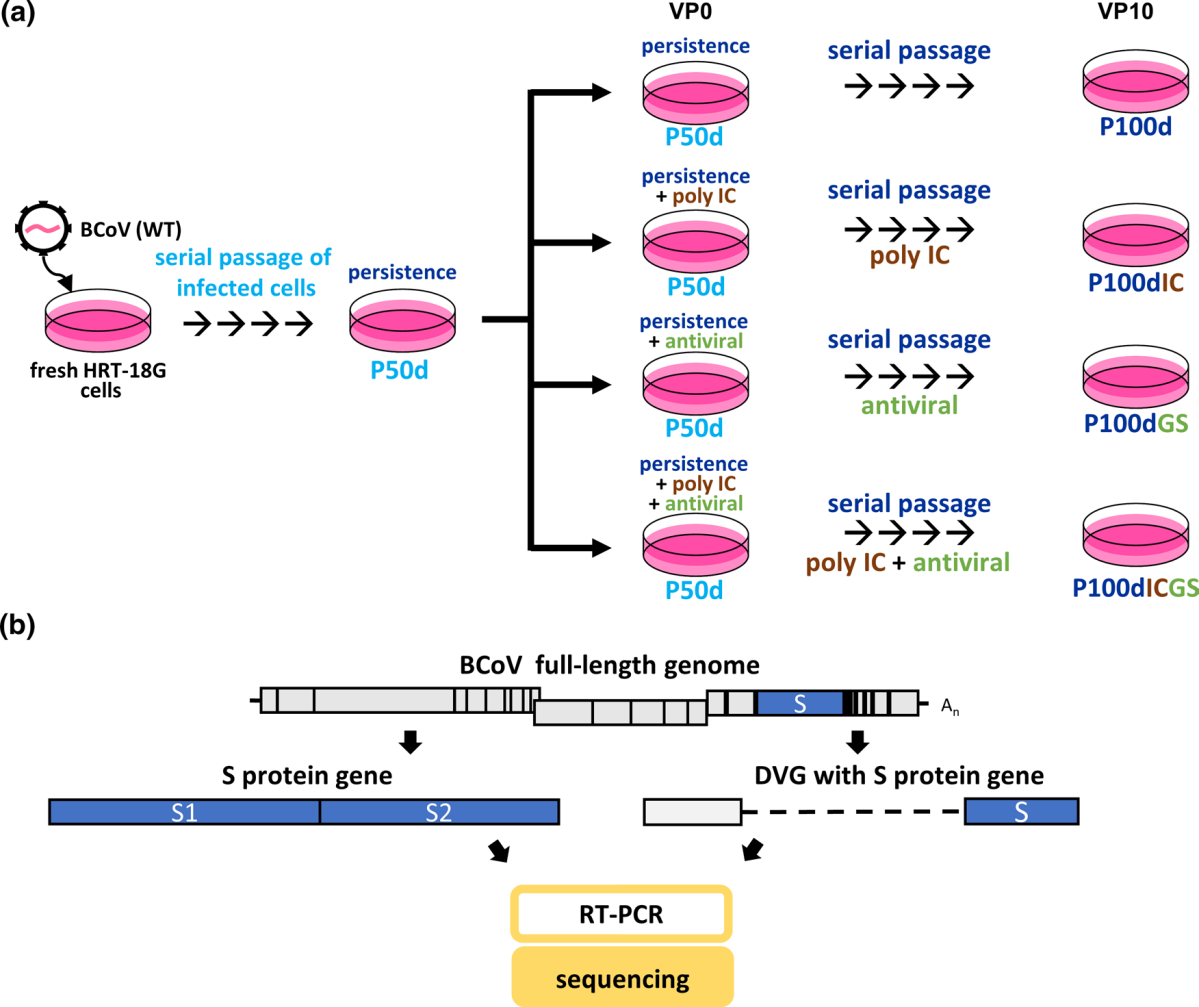
Experimental design of the study. (**a**) Fresh HRT-18G cells were infected with wild-type BCoV (WT), and the BCoV-infected cells were then passaged every 5 days. After serial passages, the viral and cellular RNA collected at 50 days of persistent infection was designated P50d (persistence at 50 days). Cells at 50 days of persistence were then left untreated or treated with poly IC or the antiviral GS-5734 or both. This stage of infection was defined as virus passage 0 (VP0). After 5 days of treatment, the cells were passaged (VP1) and were then untreated or treated with poly IC, antiviral GS-5734 or both. The step of virus passage without or with treatment of poly IC or antiviral GS-5734 or both was repeated until VP10. Cellular RNA was collected at each passage. The viral and cellular RNA collected at VP10 was designated P100d (persistence at 100 days), P100dIC (persistence and poly IC treatment at 100 days), P100dGS (persistence and antiviral GS-5734 treatment at 100 days) and P100dICGS (persistence, poly IC and antiviral GS-5734 treatment at 100 days). (**b**) Determination of the S protein sequence derived from the full-length genome and DVG by RT-PCR followed by sequencing.

Because the S protein gene can undergo mutations frequently [10, 11], it provides a better chance than other genes to identify the mutation occurring in DVGs and to evaluate the potential recombination between full-length genome and DVG. In addition, previous studies have suggested that the S protein can induce innate immunity, and the mutations also occur with high frequency in S protein genes under the treatment of poly IC-induced innate immunity or GS-5734 [34–38]. Accordingly, the S protein gene was selected as a target to investigate the evolution under multiple selection pressures for the full-length genome and DVG. The experimental design for identifying the evolutionary patterns of S protein sequences during diverse selection pressures is illustrated in Fig. 1. In brief, BCoV-infected cells at 50 days of persistence (P50d) were subjected to further persistence at 100 days (P100d) (single selection pressure), with further pressure of innate immunity (P100dIC) or an antiviral (P100GS) (double pressures), or with further pressures of both innate immunity and an antiviral (P100dICGS) (triple pressures) (Fig. 1a). Total cellular RNA was collected, followed by RT-PCR and sequencing analysis (Fig. 1b). The alterations in S protein sequences were analysed as follows.

**Fig. 2.**
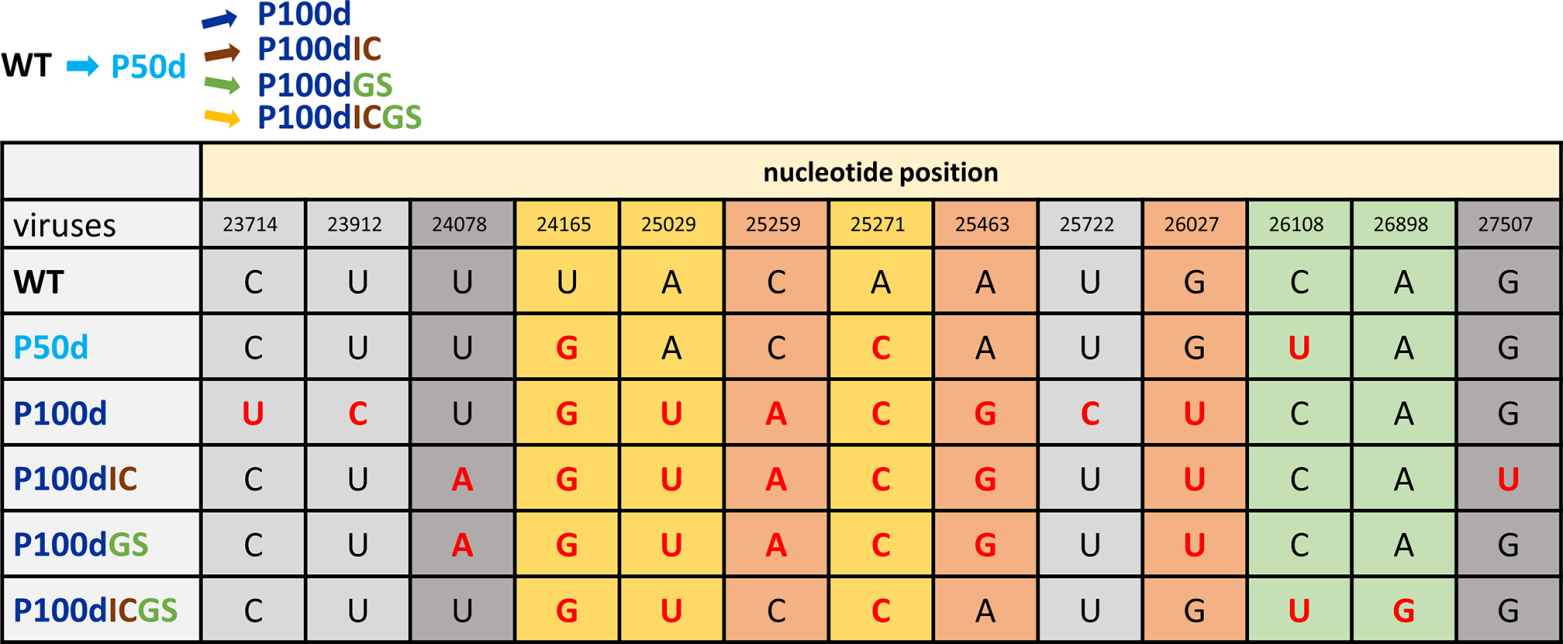
Nucleotide sequence analysis of the S protein gene in the BCoV full-length genome under diverse selection pressure(s). The mutated nucleotides are indicated in red. WT, wild-type BCoV without selection pressure; P50d, persistence at 50 days; P100d, persistence at 100 days; P100dIC, persistence and innate immunity at 100 days; P100dGS, persistence and antiviral treatment at 100 days; P100dICGS, persistence, innate immunity and antiviral treatment at 100 days.

**Fig. 3.**
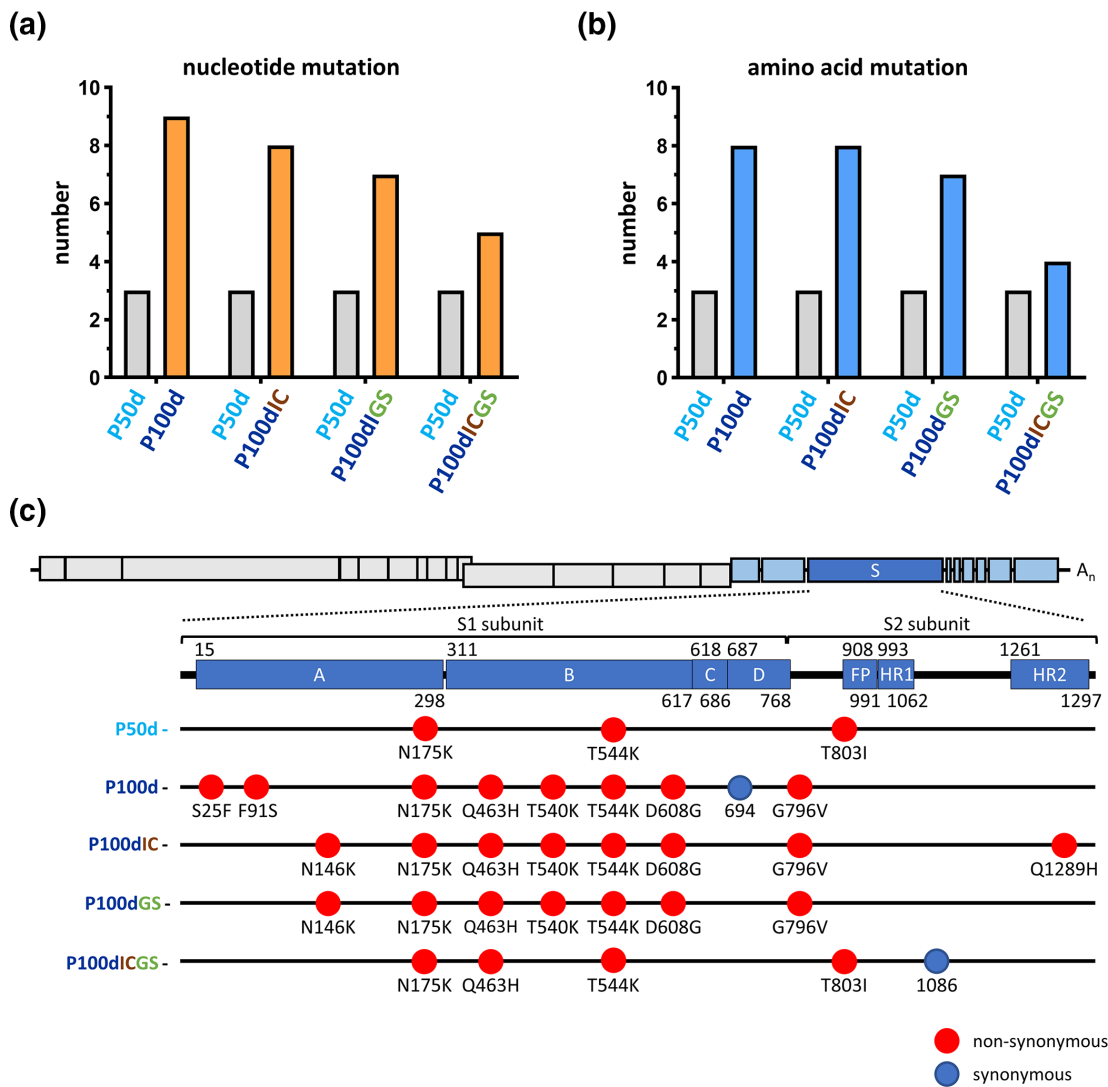
.Mutation numbers in nucleotide (**a**) and amino acid (**b**) sequences, and the distribution of the mutated amino acids in the S protein (**c**) of the full-length genome under different types of selection pressure. Amino acids with nonsynonymous or synonymous mutations in the S protein are indicated with red and blue ovals, respectively. S, spike protein; FP, fusion peptide; HR1, heptad repeat 1; HR2, heptad repeat 2.

Under the single selection pressure of persistence (P50d and P100d, Fig. 2), in comparison with the wild-type (WT) nucleotide (nt) sequence of BCoV, 3 nt substitutions were identified at 50 days of persistence. With further persistence (from 50 to 100 days of persistent infection), the number of nt substitutions (P50d and P100d, respectively, Fig. 2) increased from three to nine (Fig. 3a). Interestingly, nt 26108 (U) was substituted at 50 days of persistence but reverted to the WT sequence (C) with further persistence at 100 days (P100d). In addition, seven substituted nt at positions 23714, 23912, 25029, 25259, 25463, 25722 and 26027 may also result from further persistence at 100 days (P100d).

Under the double selection pressures of persistence and poly IC treatment (P100dIC, Fig. 2), (i) the number of nt substitutions (from P50d to P100dIC) also increased from three to eight (Fig. 3a), (ii) the nt at position 26108 (U) was substituted at 50 days of persistent infection but also reverted to the WT sequence, suggesting that the virus population with reverted sequence was selected with the additional pressure of poly IC treatment, and (iii) the additional nt substitutions at positions 24078 (A) and 27507 (U) might result from the pressure of poly IC treatment when compared with the substitutions derived from the pressure of persistence at 100 days (P100d, Fig. 2).

Similar to that under double selection pressures of persistence and innate immunity with poly IC, under the double selection pressures of persistence and antiviral GS-5734 treatment (P100dGS, Fig. 2), the number of nt substitutions also increased (from three to seven, Fig. 3A), and the nt at position 26108 (U) was also reverted to the WT sequence from 50 days of persistence (U) to 100 days of persistence and antiviral treatment (C). Additionally, the double selection pressures of persistence and antiviral drug treatment can also lead to the substitution of the nt at position 24078 (A) when compared with the substitutions derived from the pressure of persistence at 100 days (P100d) (Fig. 2).

Surprisingly, with the increased selection pressures including persistence, innate immunity with poly IC and the antiviral treatment (P100dICGS, Fig. 2), (i) the number of nt substitutions only increased from three to five (Fig. 3a) and (ii) only the nt at position 26898 (G) might specifically result from the triple pressures of persistence, innate immunity and antiviral treatment. However, in contrast to the reversion of the nt under the single pressure of persistence (P50d and P100d, Fig. 2) or double pressures of persistence and innate immunity with poly IC (P100dIC, Fig. 2) or antiviral treatment (P100dGS, Fig. 2), the nt at position 26108 (U), a substituted nt that occurred at 50 days of persistence, remained unchanged and was not reverted to the WT sequence (Fig. 2).

Regarding mutations in the amino acid sequence, the trend of the amino acid mutation number was overall similar to that for nt (Figs 3a, b and S1). In addition, most of the mutated amino acids were located in the S1 subunit of the S protein (Figs 3c and S1) under single (seven out of eight), double (six out of eight for persistence and innate immunity with poly IC; six out of seven for persistence and antiviral treatment) and triple (three out of four) selection pressure(s).

Consequently, based on the results of the S protein sequence in the full-length genome shown in Figs 1–3 and S1 and the analysis above, it is concluded that (i) the frequency of both nt and amino acid mutation is not necessarily increased with the number of selection pressures, (ii) a certain type of selection pressure(s) may lead to specific nt and thus amino acid mutations, (iii) the mutated nt can be reverted to WT nt under certain types of selection pressure, and (iv) the majority of mutations occur in the S1 subunit of the S protein.

### The altered S protein sequence can be generalized into seven patterns in terms of the dynamic alterations of certain nts during diverse selection pressures

**Fig. 4.**
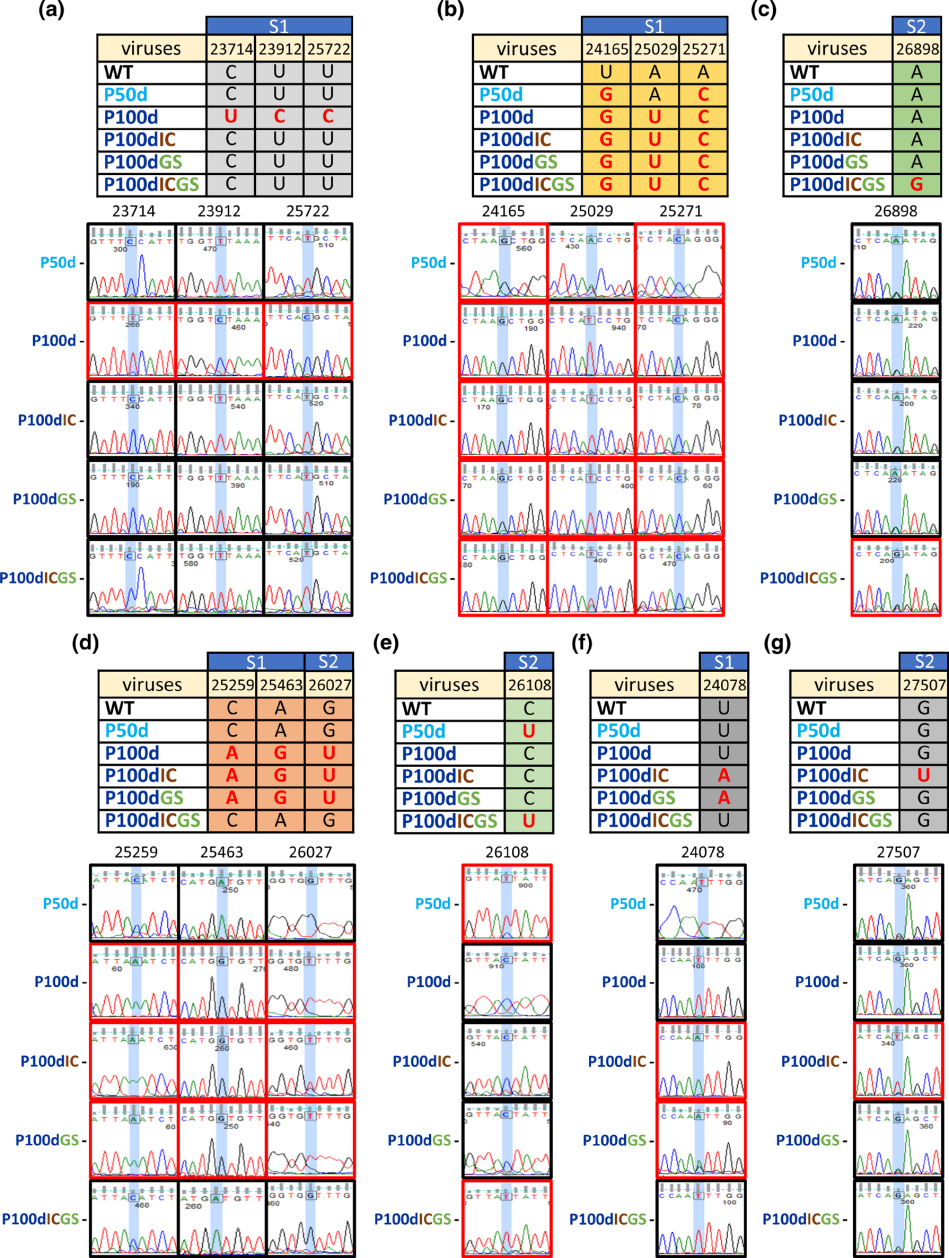
Evolutionary patterns of the S protein sequence in the full-length genome under diverse selection pressures (see text for details). (**a**) The mutation was derived from the pressure of persistent infection. (**b**) The mutation was only caused by single, double or triple selection pressure(s). (**c**) The single mutation was caused by triple selection pressures. (**d**) The mutations were caused by a single selection pressure of persistence and double selection pressures of persistence and innate immunity or antiviral treatment. (**e**) The mutation was caused by persistent infection at 50 days and triple selection pressures. (**f**) The mutation was only caused by double selection pressures of persistence and innate immunity or antiviral treatment. (**g**) The mutation was caused by double selection pressures of persistence and innate immunity.

Based on the sequential alterations of the S protein sequence under dynamic and diverse selection pressures, the altered sequence can be categorized into seven patterns, from which the potential reason for the sequence alterations can be evaluated. It was speculated that the mutations in the first pattern (Fig. 4a) may be due to the selection pressure of persistence because the mutated sequences in the first pattern do not occur under additional selection pressure(s) (persistence with innate immunity or antiviral treatment or both), suggesting that the mutated sequences may be important only under the selection pressure of persistence (P100d). In contrast, in addition to persistence, the mutated sequences in the second pattern (Fig. 4b) do appear under additional pressure(s), suggesting that the appearances of the mutations are important under these selection pressures, including persistence (P100d) and persistence with innate immunity (P100dIC) or antiviral treatment (P100dGS) or both (P100dICGS). On the other hand, the mutation in the third pattern only occurred under triple selection pressures (Fig. 4c) but did not occur under single or double selection pressure(s). Interestingly, the mutations in the fourth pattern (Fig. 4d) only occur under single or double selection pressures but not triple selection pressures. Based on the sequential nt changes during dynamic secretion pressures (from WT, P50d to P100d, P100dIC or P100dGS, Fig. 4e), it is proposed that the mutation U in P50d of the fifth pattern is due to persistence at 50 days; however, the mutation U reverts to the WT (C) under further persistence (P100d) or the additional selection pressure of innate immunity (P100IC) and antiviral treatment (P100dGS). Consequently, the nt U in P100dICGS in the fifth pattern (Fig. 4e) may occur under persistence at 50 days (P50d), but the nt U remains unchanged under the triple selection pressures of persistence, innate immunity and antiviral treatment. The mutations in the sixth and seventh patterns are simply derived from the double selection pressures of persistence and innate immunity or antiviral treatment (Fig. 4f) and the double pressures of persistence and innate immunity (Fig. 4g), respectively.

### The evolutionary features of CoV DVG under diverse selection pressure

**Fig. 5.**
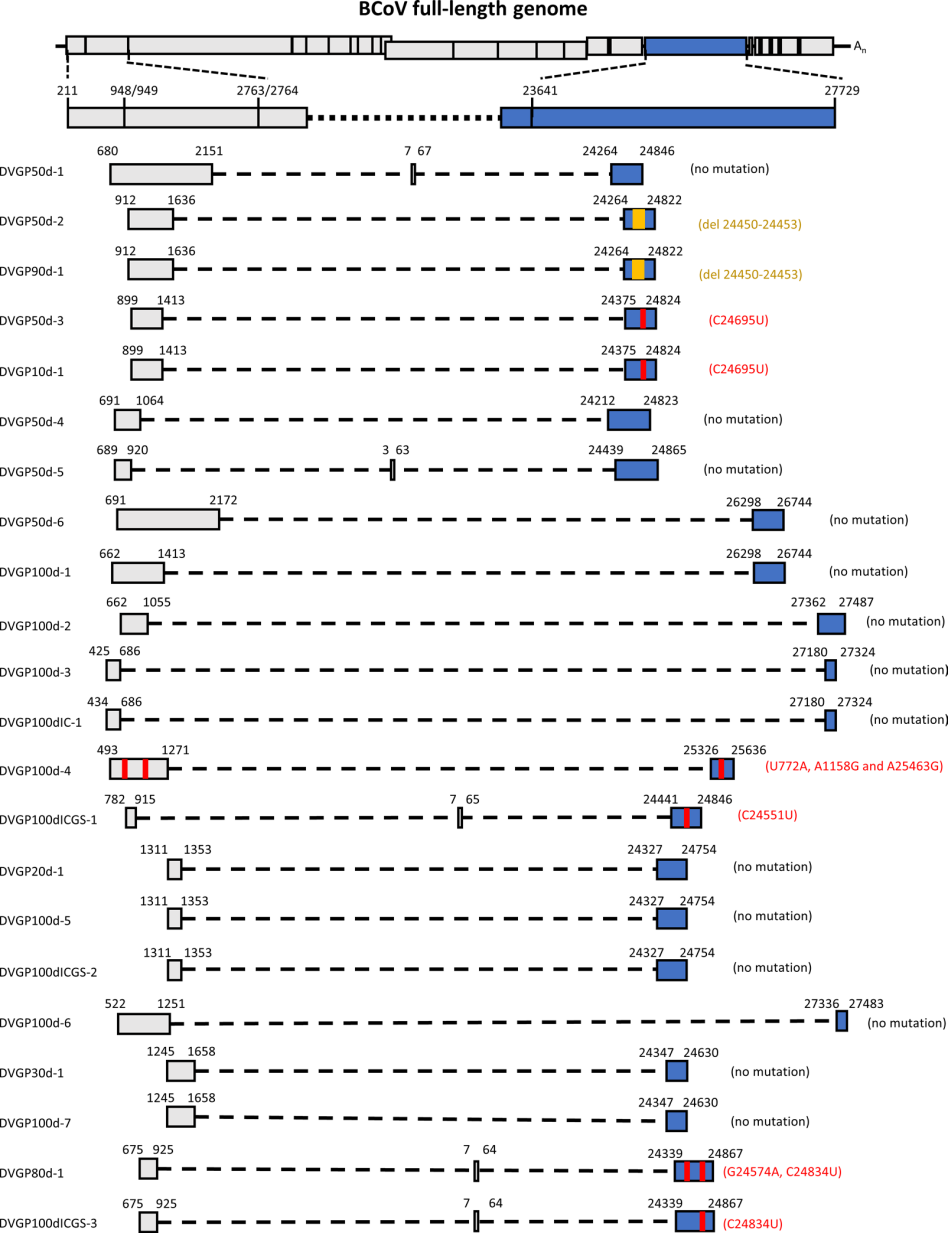
The structures of partial S protein sequence-containing BCoV DVGs identified during diverse selection pressures. Deleted or mutated nucleotides are indicated with yellow or red lines, respectively, when compared with the full-length genome. The numbers shown in each DVG structure are the nucleotide positions corresponding to the full-length genome. The dashed line indicates the truncated genome in the DVG. Numbers on both sides of the dashed line indicate the recombination positions corresponding to the full-length genome.

**Fig. 6.**
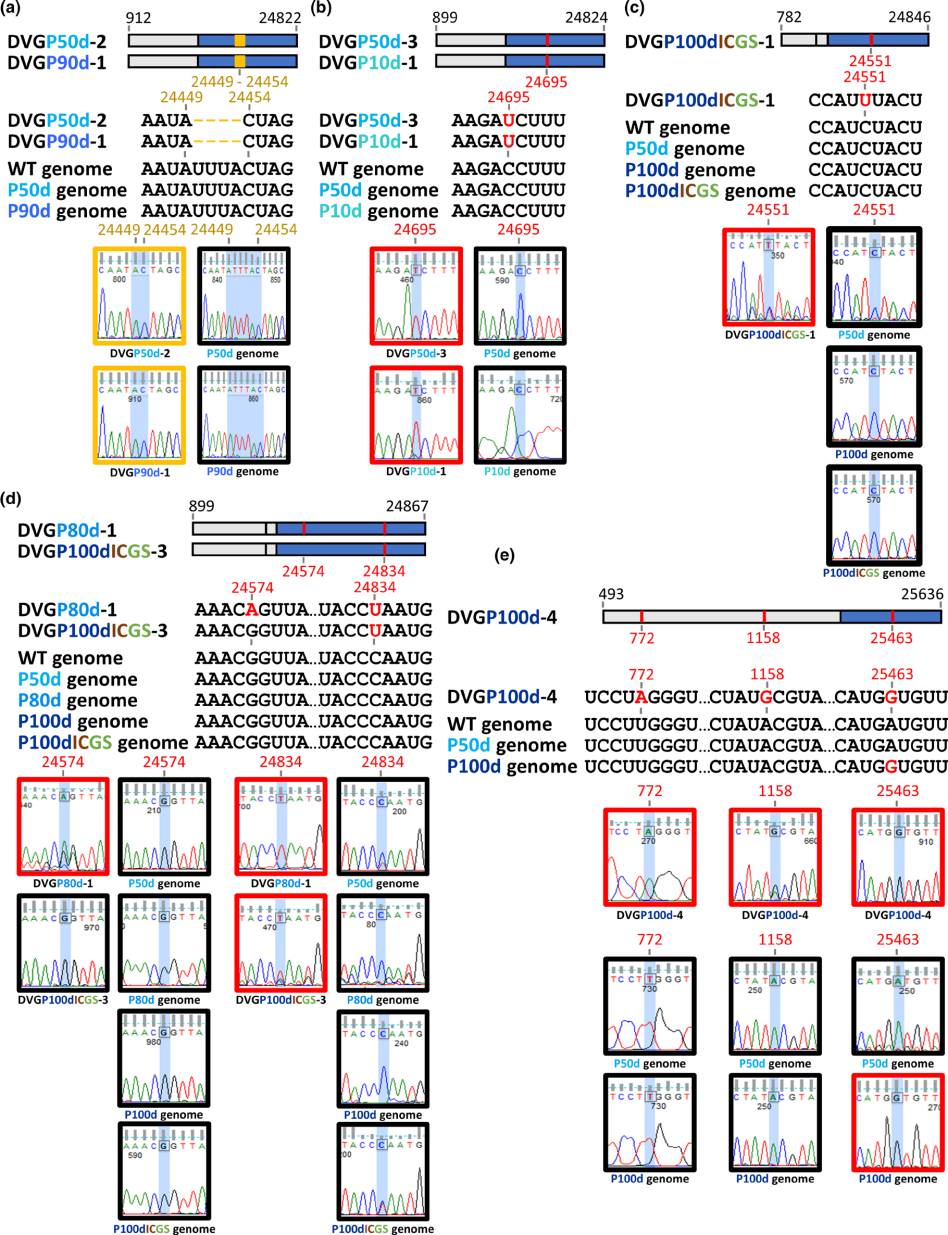
Evolutionary patterns of coronavirus S protein sequence-containing DVGs under diverse selection pressures. (**a**) The deletions identified in DVGP50d-2 and DVGP90d-1 occurred under persistent infection at 50 and 90 days, respectively. (**b**) The mutation U was detected in DVGP50d-3 and DVGP10d-1 during persistent infection at 50 and 10 days, respectively. (**c**) The mutation U was identified in DVGP100dICGS-1 under triple pressures of persistent infection, innate immunity and antiviral treatment at 100 days. (**d**) The mutations A and U and the mutation U were identified in DVGP80d-1 and DVGP100dICGS-3 under persistent infection at 80 days and triple pressures of persistent infection, innate immunity and antiviral treatment at 100 days, respectively. (**e**) Mutations A and G in the *nsp1* and *nsp2* genes, respectively, were identified in DVGP100d-4. The mutation G was identified in both the S protein gene of the full-length genome P100d and DVGP100d-4.

The DVG is a truncated version of the viral genome. In CoV, synthesis of the DVG presumably occurs through a copy-choice template-switching process. However, whether certain DVG species exist throughout the process of selection pressure and whether the structures of CoV DVGs are altered under selection pressure have not been previously studied. To investigate whether the structures of S protein sequence-containing DVGs are altered under selection pressures, total cellular RNA was collected under different selection pressures, and primer sets bound to the 5′ terminus of the full-length genome and S protein sequence were used for RT-PCR followed by sequencing analysis. The RNA species with truncated genome determined by sequencing analysis were identified as the DVG (Table 1 and Fig. 5). As summarized in Table 1, it was found that the DVG species can be detected at certain time point(s) under certain types of selection pressure, suggesting that the species of DVG are altered and thus potentially regulated during the process of dynamic and diverse selection pressures. In addition, as shown in Fig. 5, 22 DVG species with S protein sequences were identified at different time points and under different selection pressures, and among the 22 identified DVGs, eight had altered structures which are analysed in Fig. 6. It is worth to noting that, regardless of whether the DVGs contain mutations in the S protein gene, the species and amounts of the DVGs were also different under different treatment conditions (Fig. S2).

**Table 1.** DVG species are altered during selection pressure(s)

	Virus at a particular time point of selection pressure(s)^*^									
**DVG species**	**P10d**	**P20d**	**P30d**	**P50d**	**P80d**	**P90d**	**P100d**	P100dIC	P100dGS	P100dICGS
DVGP50d-1	**x**	x	x	DVG P50d-1	x	x	x	x	x	x
DVGP50d-2	x	x	x	DVG P50d-2	x	DVG P90d-1 (DVG P50d-2)	x	x	x	x
DVGP50d-3	DVG P10d-1 (DVG P50d-3)	x	x	DVG P50d-3	x	x	x	x	x	x
DVGP50d-4	x	x	x	DVG P50d-4	x	x	x	x	x	x
DVGP50d-5	x	x	x	DVG P50d-5	x	x	x	x	x	x
DVGP50d-6	x	x	x	DVG P50d-6	x	x	x	x	x	x
DVGP100d-1	x	x	x	x	x	x	DVG P100d-1	x	x	x
DVGP100d-2	x	x	x	x	x	x	DVG P100d-2	x	x	x
DVGP100d-3	x	x	x	x	x	x	DVG P100d-3	DVG P100dIC-1 (DVG P100d-3)	x	x
DVGP100d-4	x	x	x	x	x	x	DVG P100d-4	x	x	x
DVGP100d ICGS-1	x	x	x	x	x	x	x	x	x	DVG P100dICGS-1
DVGP100d-5	x	DVG P20d-1 (DVG P100d-5)	x	x	x	x	DVG P100d-5	x	x	DVG P100dICGS-2 (DVG P100d-5)
DVGP100d-6	x	x	x	x	x	x	DVG P100d-6	x	x	x
DVGP100d-7	x	x	DVG P30d-2 (DVG P100d-7)	x	x	x	DVG P100d-7	x	x	x
DVGP100d ICGS-3	x	x	x	x	DVG P80d-1 (DVG P100dICGS-3)	x	x	x	x	DVG P100dICGS-3

*DVG species with mutations are indicated in red. X indicates that the DVG species was not identified at a certain time of selection pressure or under a certain type of selection pressure.

### Evolutionary patterns of CoV S protein sequence-containing DVG under diverse selection pressures

Based on the altered sequences and the type of selection pressure, the evolutionary patterns of the S protein sequence-containing DVG at different time points under selection pressures shown in Table 1 and Fig. 5 can be classified into six patterns (Fig. 6), and are analysed as follows.

First, four deleted nts corresponding to nt 24450–24453 of the BCoV full-length genome were identified in DVGP50d-2 and DVGP90d-1 at 50 and 90 days of persistence but were not identified in the corresponding full-length genome (Fig. 6a). The results suggested that the DVG can undergo deletion under selection pressure of persistence and can evolve independently of the full-length genome.

Second, under the selection pressure of persistence at 50 days, a mutation in DVGP50d-3 was identified, corresponding to nt 24695 of the full-length genome (Fig. 6b). The mutation was also identified in DVGP10d-1 under the selection pressure of persistence at 10 days (Fig. 6b). The same mutation was not found in the full-length genome under the selection pressure of persistence at 10 and 50 days or in the WT genome. Consequently, the results suggest that (i) the mutation may occur as early as at 10 days of persistence and (ii) the DVG is not derived from the full-length genome after 10 days of persistence; thus, the DVG can evolve independently.

Third, under triple selection pressures, including persistence, innate immunity and antiviral treatment, the DVG species DVGP100dICGS-1 was identified with a mutation corresponding to nt 24551 of the full-length genome (Fig. 6c). In fact, there were two populations of DVGP100dICGS-1 under 100 days of triple selection pressures and the population with mutated nt U was slightly dominant. However, the mutation was not identified in the full-length genome at 50 and 100 days of persistence or triple selection pressure. The results therefore suggest that the DVG species DVGP100dICGS-1 identified under triple selection pressure can also evolve independently. Note that, in terms of the nt at 24549 in DVGP100dICGS-1, there were two populations of DVG, but the WT nt was the dominant population.

Fourth, the same DVG species was identified under different selection pressures: DVGP80d-1 was identified at 80 days of persistence, and DVGP100dICGS-3 was detected under triple selection pressures (DVGP100dICGS-3) (Fig. 6d). Interestingly, DVGP80d-1 contained two mutations corresponding to nt 24574 and 24834 of the full-length genome, while DVGP100dICGS-3 had only one mutation at nt 24834. In terms of nt 24574, there were two populations of DVGP80d-1, one with mutated nt A at 24574 and the other with WT nt G at 24574 at 80 days of persistence, suggesting that nt 24574 was evolving and the DVG with nt A at 24574 was the dominant population. In terms of nt at 24575, there were also two populations of DVG, but the WT nt was the dominant population. Because the mutations did not occur in the full-length genome at 80 days of persistence, the results may suggest that the same DVG species DVGP80d-1 can evolve independently. Interestingly, in terms of nt 24834 under triple selection pressures, there were almost two equal populations of the full-length genome identified in P100dICGS, one with mutated nt U and the other with WT nt C. Consequently, the mutated U at nt 24834 in DVGP100dICGS-3 may either have evolved independently or been derived from the full-length genome. Alternatively, it is also likely that the mutated U at nt 24834 in the full-length genome P100dICGS may be derived from DVGP100dICGS-3, suggesting potential recombination between the DVG and the full-length genome. Together, the results suggest that (i) the same DVG species can develop different mutations under different selection pressures and can also evolve independently and that (ii) the potential recombination between the DVG and the full-length genome can occur during evolution.

Fifth, DVGP100d-4 was only identified at 100 days of persistence, and three mutations occurred, which corresponded to nt 772, 1158 and 25463 of the full-length genome (Fig. 6e). Note that there were also two populations of DVGP100d-4, one with mutated nt G at 1158 and the other with WT nt A at 1158, suggesting that nt 1158 was evolving and DVG with nt G at 1158 was the dominant population at 100 days of persistence. Nucleotides 772 and 1158 are in the *nsp1* and *nsp2* genes, respectively, but nt 25463 is located in the S protein gene. Interestingly, mutations at nt 772 and 1158, but not at nt 25463, were not identified in the full-length genome at 100 dys of persistence (P50d). In addition, none of the three mutated nts occurred in the full-length genome at 50 days of persistence. These results suggest that the DVG may evolve independently. In line with this, there are two possibilities that can explain the origin of the mutated nt at 25463 for DVGP100d-4 and full-length genome P100d: (i) the mutated nt at 25463 in the DVG may be derived from the full-length genome; (ii) alternatively, the DVG can evolve independently, and the mutated nt at 25463 in the full-length genome may be acquired from the DVG. In either case, the results suggest potential recombination between the DVG and the full-length genome. Note that the possibility that the mutated nt at 25463 is an independent evolution product for both the DVG and full-length genome also cannot be excluded.

Sixth, the rest of the identified S protein sequence-containing DVGs did not show any mutation (Figs 5 and 6). Consequently, without markers such as mutations to speculate whether they evolved independently, they may be synthesized using the full-length genome as a template or may be replicated after synthesis with the help of the parental virus at certain time points under different selection pressures.

### Replication phenotype of the selected CoV variants under regular infection conditions

To examine whether the phenotype of the selected CoV variants was altered, the CoV variants were titrated; however, for the variants derived from the treatment of persistence and antiviral GS-5734 (P100dGS) and the treatment of persistence, poly IC and GS-5734 (P100dICGS), it was found that (i) the plaque was not detectable; (ii) the virus titre could be detected by the haemagglutination (HA) assay but was very low (the titre detected by the HA assay was 2); and (iii) the viral RNA could also be detected, but the amounts were also very low when compared with those detected from WT BCoV. Notably, the HA assay can be used for the titration of BCoV because BCoV has haemagglutinin, which can haemagglutinate erythrocytes [39–41]. In addition, the low virus titre may be due to the repeated treatment with antiviral GS-5734, leading to the very low production of virus particles. Consequently, to compare the replication efficiency of the virus variants under the same virus titre, the virus titre of 2 as determined by the HA assay for each virus variant, including WT, P50d, P100d, P100dIC, P100dGS and P100dICGS, was used for infection of fresh HRT-18 cells and the viral RNA was quantified by RT-qPCR. As shown in Fig. S3, the replication efficiency between the WT and variants P50d, P100d, P100dIC and P100dICGS showed slight differences, suggesting that, overall, the replication phenotype of the aforementioned variants was slightly altered under the regular infection conditions in fresh HRT-18 cells. However, the replication efficiency between the WT and P100dGS was significantly different, suggesting that the replication phenotype for P100dGS was significantly changed. Note that the variant P100dGS still can replicate, but the efficiency is very low. This explains why the value for P100dGS (Fig. S3) is close to zero when compared with those for WT and other variants.

## Discussion

CoVs lack proofreading capability and thus can form diverse populations of viruses or quasi-species [33, 42]. During pandemics, CoVs can face selection pressures such as persistence, immune responses and antiviral treatment and thus may evolve over time with increased viral fitness. The alterations in genome structure, including the full-length genome and DVG, under dynamic or multiple selection pressures in CoVs have not been experimentally studied. Experiments applying dynamic and multiple selection pressures allow us to identify the sequential changes in nt sequences and thus the evolutionary trends. Consequently, the relative mutation number between diverse selection pressures and the mutated nt derived from the full-length genome or DVG under specific selection pressure(s) at certain time points can then be identified.

CoVs are prone to mutation during replication due to the lack of proofreading capability in their RNA polymerases, leading to a diverse population of viruses or quasi-species [33, 42]. Thus, the genome structures of CoV in infected cells may contain a diverse population of variants and a single master sequence [43, 44]. Accordingly, under environmental challenges such as the selection pressures in the current study, due to constant mutation and selection, quasi-species populations are shifted in environments under different selection pressures, and the main population of the virus is selected, leading to the specific mutations identified in this study. It is speculated that the selected mutations may contribute to the synthesis of a new phenotype for the virus to rapidly adapt to the new environment, suggesting positive evolution. In light of function-oriented selection, it remains unclear why different selection pressures led to a high intensity of mutation at the same target (S protein). It is speculated that, to adapt to a variety of host cells for survival, the coronavirus S protein-encoding gene has evolved to contain different mutations in the virus population during infection; this has led to sequence diversity, thus enabling rapid adaptation to environmental challenges. While not in the scope of this study, functional studies related to mutations observed in the current experiments may help to identify candidate amino acid residues that are responsible for CoV adaptation under different selection pressures. Additionally, future studies related to whether the selected virus variants can develop resistance against antivirals may also contribute to the development of antiviral strategies for CoVs.

In this study, unexpectedly, the number of mutations under a single selection pressure was higher than that under triple selection pressure (Fig 3a, b). Moreover, among the diverse mutation patterns, the reversion of the nt sequence to the WT is also selected under further selection pressure (Fig. 4E). The mechanism leading to this outcome remains unclear. However, it suggests that appropriate mutations but not an increased number of mutations may be the best strategy for viruses to weather a harsh environment. This argument is supported by the finding that certain types of mutation patterns have a tendency to remain unchanged under triple selection pressures (Fig. 4d). The potential reversion of the nt sequence to the WT is also found among SARS-CoV-2 variants (Fig. S4) [45, 46] in terms of the timeline of variant emergence. However, unlike the experimental system used in the current study in which the time, environment and selection pressures are controlled and the sequential evolution process can be monitored, it remains unclear whether the mutation reversal among SARS-CoV-2 variants is derived from a sequential evolution process or is an independent evolutionary event that occurred in a certain geographical region from a certain SARS-CoV-2 variant. Although it is a challenge to predict the evolutionary trend of CoVs during natural pandemics, the results obtained from the experimental system allow us to identify mutation patterns under diverse selection pressures and thus may provide information to explain the naturally occurring evolutionary trend of CoV during pandemics.

The evolutionary patterns of CoV DVGs under selection pressure have not been previously studied. It is suggested in the current study that CoV DVG species (i) can appear and then disappear or can appear, disappear and then reappear at a certain time point under the selection pressure of persistence or (ii) appear only under certain selection pressure(s) (Table 1). The results also raise the question of why DVGs with or without mutation can be identified but disappear at a certain time during selection pressures. If the DVG is derived from the full-length genome and cannot replicate and be packaged, it is possible that the DVG may be synthesized at a certain time point under certain selection pressures due to the regulation of viral gene expression. On the other hand, once a certain DVG species is synthesized, the DVG may not truly disappear if it can replicate and be packaged. However, the amounts of the DVG may be altered at certain times during the process of certain selection pressures, leading to detectable and undetectable DVG species, as shown in Table 1. Consistently, such alterations in terms of the synthesized amounts of DVG and thus diverse evolutionary patterns shown in Table 1 may be one of the strategies by which CoVs adapt to dynamic environments for viral fitness at certain time points under certain or diverse selection pressures.

**Fig. 7.**
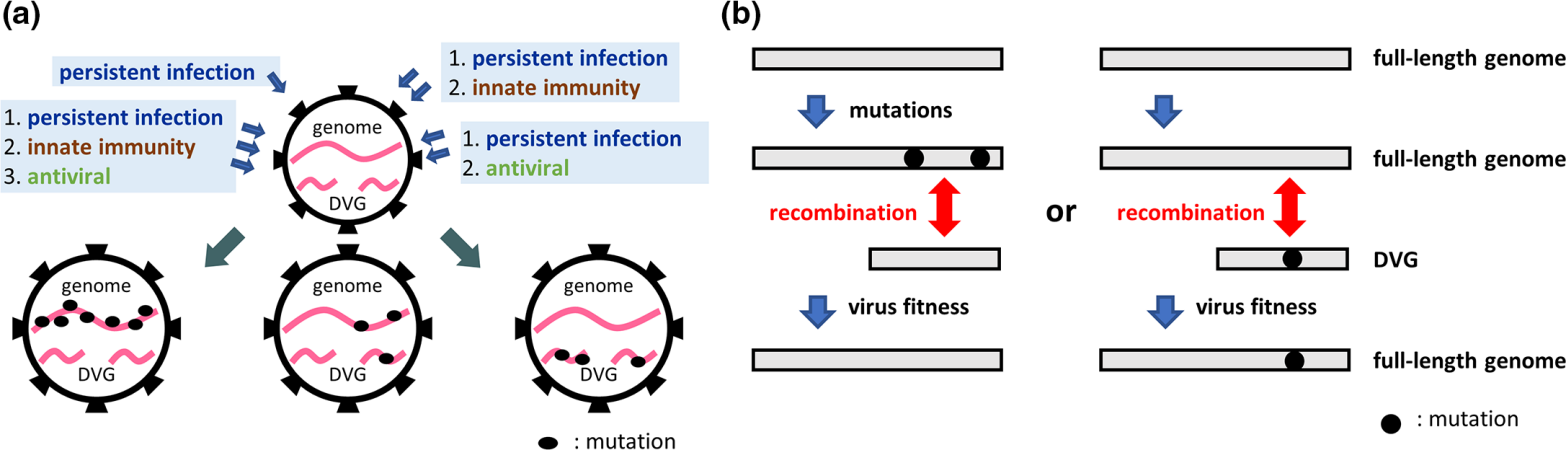
Potential biological significance of DVG evolution. (**a**) The full-length genome and DVG are able to evolve independently under diverse selection pressures. (**b**) Potential recombination between the full-length viral genome and DVG may enable coronaviruses to overcome catastrophic errors for viral fitness.

It was also suggested for the first time in this study that the distinct populations of CoV genomes, such as the full-length genome and DVG in infected cells, can undergo mutations and evolve independently. Since recombination between CoV RNA species can occur during natural infections [47, 48], and the full-length genome and DVG can evolve independently, in addition to the mutations occurring in the full-length genome, recombination between the DVG and the error-prone full-length genome may assist CoVs in overcoming catastrophic error and thus restoring the fitness of the genome under selection pressures. The potential evidence for the recombination event is shown in Fig. 6d, e, in which the recombination may potentially occur based on the common mutations identified both in the DVG and full-length genome. In fact, the deletion and mutations identified in the DVGs as shown in Fig. 6 all can alter the amino acid sequence of nsp1 and S protein. Once the recombination between the DVG and full-length genome occurs, the full-length genome can acquire the deletion or mutations from the DVG and the biological characteristics may be altered, affecting virus fitness. In summary, it is argued that (Fig. 7), in addition to conventional mutations in the full-length genome, the synthesis of various types of DVG species, with or without mutations (Fig. 7a), and recombination between mutated or unmutated DVGs and the full-length genome (Fig. 7b) are also means by which CoVs combat altered environments such as selection pressures. On the other hand, the DVGs selected under selection pressures may develop resistance against selection pressures such as antiviral drugs and vaccines. Once the selected DVG recombines with the full genome, the resulting CoV variants may have the potential to escape from antivirals and immune response surveillance. Consequently, the selected virus with resistance may survive and repopulate within hosts or transmit to other hosts, posing an threat to public health. The present study suggests that the dynamic evolutionary patterns of the S protein sequence can occur in the full-length genome and DVG and that there is recombination potential between full-length genomes under diverse selection pressures *in vitro*. Notably, two independent experiments were performed in the current study, and the mutations of the S protein-encoding gene in the full-length genome and DVGs were all identified in the two independent experiments although the mutation sites were different.

The current study suggested that under the selection pressures of persistence (P50d and P100d), persistence and poly IC-induced innate immunity (P100dIC) or antiviral GS-5734, persistence and poly IC-induced innate immunity (P100dICGS), the replication phenotype of the selected variants was, overall, slightly changed under the regular infection conditions in comparison with the WT (Fig. S3). However, under selection pressure of antiviral GS-5734 and persistence (P100dGS), the replication phenotype was significantly altered in terms of replication efficiency. The function of antiviral GS-5734 is to block virus replication; therefore, it is speculated that the mutations in replicase genes may occur during selection pressures, leading to alterations in the replication phenotype (Fig. S3). However, because the focus of the study was the S protein-encoding gene, whether the mutations in replicase or other genes in the genome are responsible for the alteration of phenotype remains unclear. Future studies on the identification of mutations in the entire genome, including replicase genes, may provide clues to explain why the replication phenotype of the variant is altered under regular infection conditions.

In summary, in this study, we employed BCoV as a test model to explore how a CoV reacted to multiple selection pressures in terms of the S protein gene in the full-length genome and DVG. We identified the evolutionary patterns of the S protein gene in the full-length genome and DVG under dynamic and diverse election pressures. Furthermore, it is also suggested for the first time that (i) distinct populations of CoV genomes, including the full-length genome and DVG in infected cells, can undergo mutations and evolve independently, and (ii) there is potential for recombination to occur between the full-length genome and DVG. Finally, although the results may provide information on the possible evolutionary patterns during diverse selection pressures, the current *in vitro* study can indicate only that CoVs may bear the aforementioned evolutionary characteristics but cannot reflect what actually occurs *in vivo*. This is because, in addition to the diverse selection pressures, other factors, including host immune responses, intra- and interhost virus fitness, and viral transmissibility and stability, may also lead to the different outcomes of evolutionary patterns in the S protein-encoding gene *in vivo*.

## Supplementary Data

Supplementary material 1
