# Supplementary material for: Evolution of the coronavirus spike protein in the full-length genome and defective viral genome under diverse selection pressures
Source: J Gen Virol. 2023 Nov 24;104(11):001920. doi: 10.1099/jgv.0.001920 (PMC10768696; doi:10.1099/jgv.0.001920)
Supplement: Supplementary material 1 [file jgv-104-1920-s001.pdf]

Supplementary Materials for

The evolution of the coronavirus spike protein in the full-length genome and defective viral genome under diverse selection pressures

Ching-Hung Lin[1a], Hon-Man-Herman Tam[2a], Cheng-Yao Yang[1], Feng-Cheng Hsieh[1], Jiun-Long Wang[3,4,5], Chun-Chun Yang[1], Hsuan-Wei Hsu[1], Hao-Ping Liu[2*] and Hung-Yi Wu[1*]

a: Equal contributors

1 Graduate Institute of Veterinary Pathobiology, College of Veterinary Medicine, National Chung Hsing University, Taichung 40227, Taiwan

2 Department of Veterinary Medicine, College of Veterinary Medicine, National Chung Hsing University, Taichung 40227, Taiwan

3 Division of Chest Medicine, Department of Internal Medicine, Taichung Veterans General Hospital, Taichung 40705, Taiwan

4 Department of Post-Baccalaureate Medicine, College of Medicine, National Chung Hsing University, Taichung 40227, Taiwan

5 Department of Life Sciences, National Chung Hsing University, Taichung 40227, Taiwan

*Corresponding Author

Corresponding Footnote: Graduate Institute of Veterinary Pathobiology and Department of Veterinary Medicine, College of Veterinary Medicine, National Chung Hsing University, Taichung 40227, Taiwan

Telephone: 886-4-22840369; Fax: 886-4-22862073

Email: hwu2@dragon.nchu.edu.tw; hpliu@dragon.nchu.edu.tw

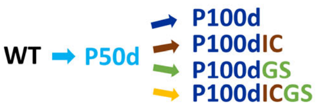

	a. a. position												
viruses	25	91	146	175	463	540	544	608	694	796	803	1086	1289
**WT**	S	F	N	N	Q	T	T	D	H	G	T	Q	Q
**P50d**	S	F	N	K	Q	T	K	D	H	G	I	Q	Q
**P100d**	F	S	N	K	H	K	K	G	H	V	T	Q	Q
**P100dIC**	S	F	K	K	H	K	K	G	H	V	T	Q	H
**P100dGS**	S	F	K	K	H	K	K	G	H	V	T	Q	Q
**P100dICGS**	S	F	N	K	H	T	K	D	H	G	I	Q	Q

Fig. S1. Amino acid sequence analysis of the S protein gene in the BCoV full-length genome under diverse selection pressure(s). The mutated amino acids are indicated in red. WT, wild-type BCoV without selection pressure; P50d, persistence at 50 d; P100d, persistence at 100 d; P100dIC, persistence and innate immunity treatment at 100 d; P100dGS, persistence and antiviral treatment at 100 d; P100dICGS, persistence, innate immunity and antiviral treatment at 100 d. a.a., amino acid.

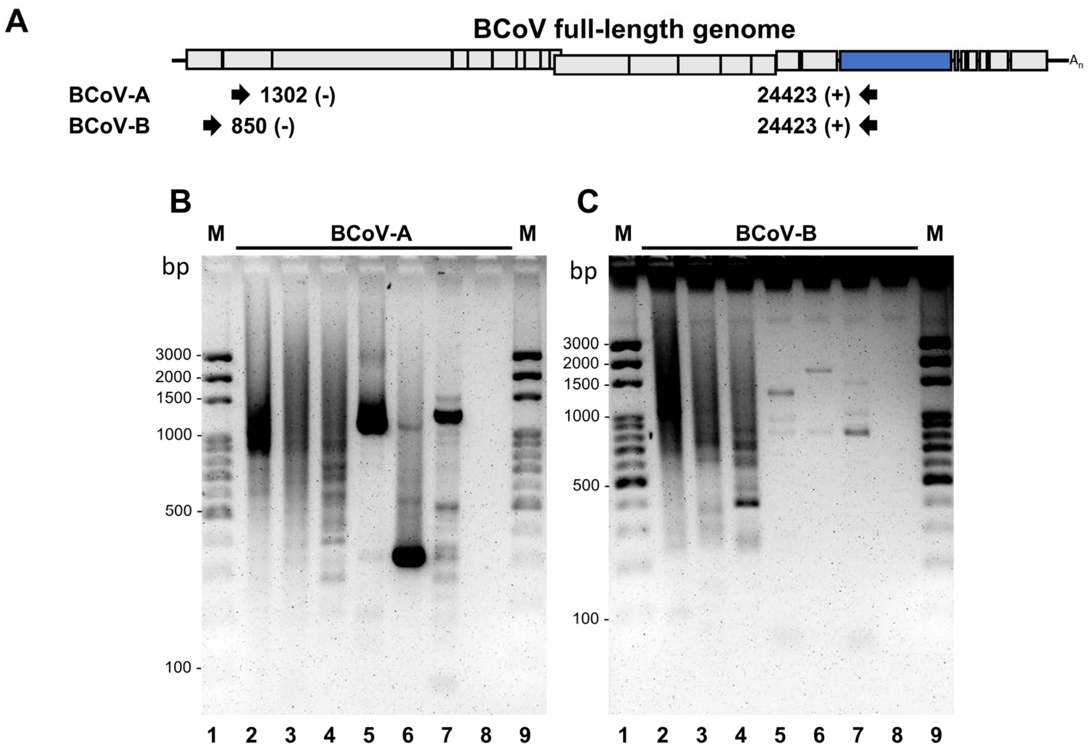

Fig. S2. The species and amounts of DVGs are altered under different selection pressures. **(A)** Diagram depicting the primer sets used for determining the synthesis of BCoV DVGs in Figs. (B)-(C)**. (B)-(C)** Detection of BCoV DVG synthesis under different selection pressures by RT-PCR. Lanes 1 and 9: DNA size marker; Lane 2: wild-type BCoV without selection pressure; Lane 3: P50d, persistence at 50 d; Lane 4: P100d, persistence at 100 d; Lane 5: P100dIC, persistence and innate immunity treatment at 100 d; Lane 6: P100dGS, persistence and antiviral treatment at 100 d; Lane 7: P100dICGS, persistence, innate immunity and antiviral treatment at 100 d: Lane 8: mock infection. bp, base-pair; M, DNA size marker.

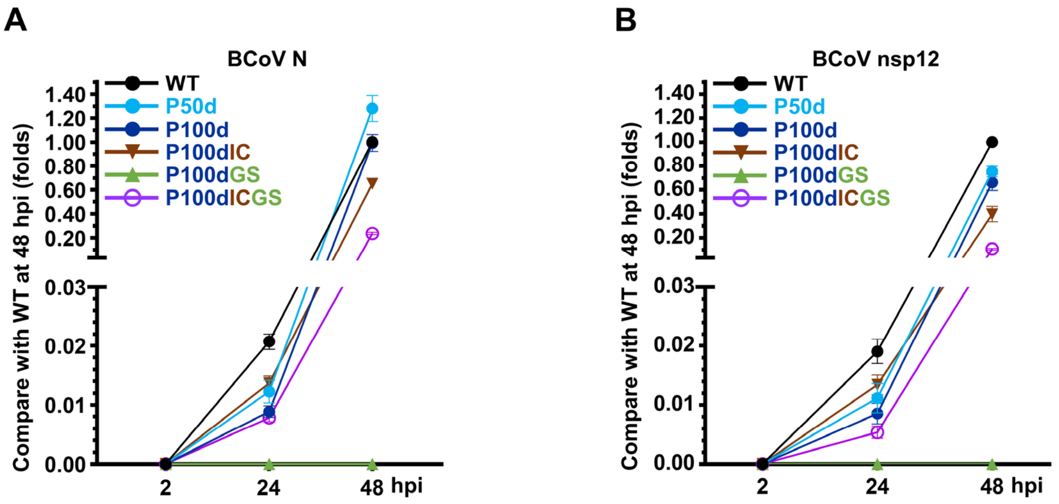

Fig. S3. The relative replication efficiency between the WT and variants, including P50d, P100d, P100dIC, P100dGS and P100dICGS under regular infection conditions, as quantitated by RT–qPCR for N protein (A) and nsp12 genes (B). hpi, hours postinfection. The values represent the means ± SDs from three individual experiments. ***$p < 0.001$.

	S1														
	a. a. position														
**virus strains**	18	19	20	24	25	26	27	67	69	70	80	83	95	138	142
Wuhan	L	T	T	L	P	P	A	A	H	V	D	V	T	D	G
Alpha	L	T	T	L	P	P	A	A	**del**	**del**	D	V	T	D	G
Beta	L	T	T	L	P	P	A	A	H	V	**A**	V	T	D	G
Gamma	**F**	T	**N**	L	P	**S**	A	A	H	V	D	V	T	**Y**	G
Delta	L	**R**	T	L	P	P	A	A	H	V	D	V	T	D	G
Omicron BA.1	L	**T**	T	L	P	P	A	**V**	**del**	**del**	D	V	**I**	D	**D**
Omicron BA.2.75	L	**I**	T	**del**	**del**	**del**	**S**	A	H	V	D	**A**	T	D	**D**
Omicron XXB.1.5	L	**I**	T	**del**	**del**	**del**	**S**	A	H	V	D	V	T	D	**D**
Omicron BA.5	L	**I**	T	**del**	**del**	**del**	**S**	A	**del**	**del**	D	V	T	D	**D**
Omicron BQ1.1	L	**I**	T	**del**	**del**	**del**	**S**	A	**del**	**del**	D	V	T	D	**D**

	S1														
	a. a. position														
**virus strains**	143	144	145	146	147	152	156	157	158	183	190	210	211	212	213
Wuhan	V	Y	Y	H	K	W	E	F	F	Q	R	I	N	L	V
Alpha	V	**del**	Y	H	K	W	E	F	F	Q	R	I	N	L	V
Beta	V	Y	Y	H	K	W	E	F	F	Q	R	I	N	L	V
Gamma	V	Y	Y	H	K	W	E	F	F	Q	**S**	I	N	L	V
Delta	V	Y	Y	H	K	W	**del**	**del**	**G**	Q	R	I	N	L	V
Omicron BA.1	**del**	**del**	**del**	H	K	W	E	F	F	Q	R	I	**del**	**I**	V
Omicron BA.2.75	V	Y	Y	H	**E**	**R**	E	**L**	F	**E**	R	**V**	N	L	**G**
Omicron XXB.1.5	V	**del**	Y	**Q**	K	W	E	F	F	Q	R	I	N	L	**E**
Omicron BA.5	V	Y	Y	H	K	W	E	F	F	Q	R	I	N	L	**G**
Omicron BQ1.1	V	Y	Y	H	K	W	E	F	F	Q	R	I	N	L	**G**

	S1														
	a. a. position														
**virus strains**	252	275	339	346	368	371	373	375	376	405	408	417	440	444	445
Wuhan	G	G	G	R	L	S	S	S	T	D	R	K	N	K	V
Alpha	G	G	G	R	L	S	S	S	T	D	R	K	N	K	V
Beta	G	G	G	R	L	S	S	S	T	D	R	N	N	K	V
Gamma	G	G	G	R	L	S	S	S	T	D	R	**N/T**	N	K	V
Delta	G	G	G	R	L	S	S	S	T	D	R	K	N	K	V
Omicron BA.1	G	G	**D**	R	L	**L**	**P**	**F**	T	D	R	**N**	**K**	K	V
Omicron BA.2.75	G	**S**	**H**	R	L	**F**	**P**	**F**	**A**	**N**	**S**	**N**	**K**	K	V
Omicron XXB.1.5	**V**	G	**H**	**T**	**I**	**F**	**P**	**F**	**A**	**N**	**S**	**N**	**K**	K	**P**
Omicron BA.5	G	G	**D**	R	L	**F**	**P**	**F**	**A**	**N**	**S**	**N**	**K**	K	V
Omicron BQ1.1	G	G	**D**	**T**	L	**F**	**P**	**F**	**A**	**N**	**S**	**N**	**K**	**T**	V

	S1														
	a. a. position														
**virus strains**	446	452	460	478	484	486	490	493	496	498	501	505	547	570	614
Wuhan	G	L	N	T	E	F	F	Q	G	Q	N	Y	T	A	D
Alpha	G	L	N	T	E	F	F	Q	G	Q	Y	Y	T	**D**	**G**
Beta	G	L	N	T	K	F	F	Q	G	Q	Y	Y	T	A	**G**
Gamma	G	L	N	T	K	F	F	Q	G	Q	Y	Y	T	A	**G**
Delta	G	**R**	N	**K**	E	F	F	Q	G	Q	**N**	Y	T	A	**G**
Omicron BA.1	**S**	L	N	**K**	**A**	F	F	**R**	**S**	**R**	Y	**H**	**K**	A	**G**
Omicron BA.2.75	**S**	L	**K**	**K**	**A**	F	F	**Q**	**G**	**R**	Y	**H**	T	A	**G**
Omicron XXB.1.5	**S**	L	**K**	**K**	**A**	**P**	**S**	**Q**	**G**	**R**	Y	**H**	T	A	**G**
Omicron BA.5	G	**R**	N	**K**	**A**	**V**	F	**Q**	**G**	**R**	Y	**H**	T	A	**G**
Omicron BQ1.1	G	**R**	**K**	**K**	**A**	**V**	F	**Q**	**G**	**R**	Y	H	T	A	**G**

	S1			S2												
	a. a. position			a. a. position												
**virus strains**	655	679	681	701	716	764	796	856	950	954	969	981	982	1027	1118	1176
Wuhan	H	N	P	A	T	N	D	N	D	Q	N	L	S	T	D	V
Alpha	H	N	**H**	A	**I**	N	D	N	D	Q	N	L	**A**	T	**H**	V
Beta	H	N	P	**V**	T	N	D	N	D	Q	N	L	S	T	D	V
Gamma	**Y**	N	P	A	T	N	D	N	D	Q	N	L	S	**I**	D	**F**
Delta	H	N	**R**	A	T	N	D	N	**N**	Q	N	**F**	S	T	D	V
Omicron BA.1	**Y**	**K**	**H**	A	T	**K**	**Y**	**K**	D	**H**	**K**	L	S	T	D	V
Omicron BA.2.75	**Y**	**K**	**H**	A	T	**K**	**Y**	N	D	**H**	**K**	L	S	T	D	V
Omicron XXB.1.5	**Y**	**K**	**H**	A	T	**K**	**Y**	N	D	**H**	K	L	S	T	D	V
Omicron BA.5	**Y**	**K**	**H**	A	T	**K**	**Y**	N	D	**H**	K	L	S	T	D	V
Omicron BQ1.1	**Y**	**K**	**H**	A	T	**K**	**Y**	N	D	**H**	**K**	L	S	T	D	V

Fig. S4. Sequencing analysis of the S protein in SARS-CoV-2 full-length genome during pandemics. SARS-CoV-2 reference genomes: Wuhan, GenBank: NC_045512.2; Alpha B.1.1.7,

GenBank: QWE88920.1; Beta B.1.351, GenBank: QRN78347.1; Gamma P.1, GenBank: QRX39425.1; Delta B.1.617.2, GenBank: QUD52764.1; Omicron BA.1, GenBank: UFO69279.1; Omicron BA.2.75, GenBank: UTM82166.1; Omicron XBB.1.5, GenBank: UZG29433.1; Omicron BA.5, GenBank: UOZ45804.1; Omicron BQ.1.1, GenBank: UWM38596.1). Emergence: Alpha B.1.1.7 (UK, Sep 2020); Beta, B.1.351 (South Africa, Aug 2020); Gamma P.1 (Brazil, Jul 2020); Delta B.1.617.2 (India, Dec 2020); Omicron BA.1 (South Africa, Dec 2021); Omicron BA.2.75 (India, spring 2022); Omicron XBB.1.5 (USA, Nov 2022); Omicron BA.5 (South Africa, Jan 2022); BQ.1.1 (Nigeria, Jul 2022).

Table S1. Oligonucleotides used in the study

Oligonucleotides[a]	Polarity[b]	Sequence (5' to 3')
BCV 809(+)	—	cgtttatgatctttattgtagg
BCV 1050(+)	—	gctccaaataggaaataatgcc
BCV 1365(+)	—	cttgttcgcagtcaatataaccaaactgc
BCV 1637(+)	—	cgtgctggactccaatacac
BCV 1992(+)	—	caaataatatgcgcgcggaacc
BCV 3114(+)	—	gtcatcatcttcaggagctg
BCV 21000(+)	—	gtactcccctatgttcttag
BCV 21637(+)	—	gcaagactaatgtgcggcgctg
BCV 24146(+)	—	ccaactgtgatttacatgc
BCV 24239(+)	—	gtaatcagcattcacatcatatg
BCV 24286(+)	—	gcataaaaagtaccacc
BCV 24423(+)	—	ccaatattctaaagtcatagc
BCV 24556(+)	—	cgtttaattcataaacacc
BCV 24648(+)	—	gcaccgacttatcattaagc
BCV 24778(+)	—	cacataccatatatcttagc
BCV 25229(+)	—	cattgggcagcattatggcaag
BCV 25753(+)	—	cgtaattgcatttaatattccg
BCV 26047(+)	—	cctatagtaaactctgaagg
BCV 26298(+)	—	cgtctacattgaaattaacg
BCV 26548(+)	—	ggtggcagccaaagtgtatcc
BCV 26908(+)	—	gtctgtctatctgagcttgcgcttc
BCV 27038(+)	—	ccattaccacaaaaatttatcc
BCV 27853(+)	—	gttatataagacacagtacccac
BCV 27979(+)	—	cagtggtgctagtgtcaatacc
BCV END 2(+)	—	gtgattcttccaattggccataattaacttc

a The positive symbols in the oligonucleotide names indicate the polarities of the nucleic acids to which the oligonucleotides anneal.
b Polarity of the oligonucleotide relative to the positive-strand viral genome.

Table S1. Oligonucleotides used in the study (*Continued*)

Oligonucleotides[a]	Polarity[b]	Sequence (5' to 3')
BCV 20(-)	+	gattgtgagcgatttgcgtgc
BCV 7-32(-)	+	gagcgatttgcgtgcgtgcatcccgcttcact
BCV 21-51(-)	+	cgtgcatcccgcttcactgatctcttgttag
BCV 39(-)	+	gatctcttgttagatctttttataatctaaacttta
BCV 81(-)	+	catccactccctgtattctatgcttgtggg
BCV 126(-)	+	gtggtgtctatattcatttc
BCV 157(-)	+	gctttcagccagggacgtgttgtatcctaggc
BCV 426(-)	+	gcagtctagcctaatacgtg
BCV 443 (-)	+	gtgaaattgttatgaatacacg
BCV 450(-)	+	tgttatgaatacacgtccatatg
BCV 500(-)	+	tgcagtcccgcgaagcagttttgg
BCV 550(-)	+	gaggcatgctatgtgagaggttgt
BCV 577(-)	+	cctaatggatggaccatgggtttg
BCV 590(-)	+	ccatgggtttgtttcggcgtagaa
BCV 628(-)	+	ggtcgttgcgctgttaacaagc
BCV 650(-)	+	atgtggcctatcagctatatatga
BCV 679(-)	+	cctgcgggtgtctgttttgg
BCV 700(-)	+	gcaggtcaatttgtgggttgggtt
BCV 750(-)	+	atcccggaaatttattgttccttg
BCV 770(-)	+	cttgggttatgtacttgcgtaagt
BCV 780(-)	+	gtacttgcgtaagtgtggcgaaaa
BCV 850(-)	+	tataattttaaagttgaggatgct
BCV 862(-)	+	gttgaggatgcttacgacctg
BCV 879(-)	+	cctggttcatgatgagcctaag
BCV 900(-)	+	gggtaagttttctaagaaggctta
BCV 950(-)	+	ttaaaccgcttctctatgtagacc
BCV 979(-)	+	ggttgtgattatactggtgg
BCV 1114(-)	+	cgtgatccacgttatgttatg
BCV 1159(-)	+	cgtagtgttgcatatgttgc
BCV 1246(-)	+	gactctattattttacgtcaac
BCV 1302(-)	+	ggaggcagatgcagttgtaaatgc
BCV 1365(-)	+	gcagtttggttatattgactgc

a The negative symbols in the oligonucleotide names indicate the polarities of the nucleic acids to which the oligonucleotides anneal.
b Polarity of the oligonucleotide relative to the positive-strand viral genome.

Table S1. Oligonucleotides used in the study (*Continued*)

Oligonucleotides[a]	Polarity[b]	Sequence (5' to 3')
BCV 1402(-)	+	gactgcgaacaagacttgtgtg
BCV 1536(-)	+	gagtgcagcaggttatggtgg
BCV 2002(-)	+	ggtcaagcattttgtgactacg
BCV 23341(-)	+	ggatgctggttttactagc
BCV 23415(-)	+	ggtatgataatgttagcagtg
BCV 23498(-)	+	cctatttgtgtgtatgatcc
BCV 23730(-)	+	caccggtgctccttctattagcac
BCV 23953(-)	+	gctaaggtcaaaaataccaagg
BCV 24270(-)	+	ccatttttatcaagaaggtgg
BCV 24607(-)	+	cgacgtatacctaatcttcc
BCV 24647(-)	+	ggcttaatgataagtcggtgccctc
BCV 24775(-)	+	gctgctaagatatatggtatg
BCV 25171(-)	+	ggtataggcacttgtcctgc
BCV 25310(-)	+	gcataggtgagcactgttcgg
BCV 25543(-)	+	gatctttatggtattacagg
BCV 26026(-)	+	ggtttgtatgaaattcaaatacc
BCV 26051(-)	+	cagagtttactataggtaatatggagg
BCV 26650(-)	+	gtgttaccatggatgtgttaagtc
BCV 26710(-)	+	gctcttgatgctattcaggaagg
BCV 27128(-)	+	ctactaagtatgtcactgcg
BCV 27505(-)	+	cagagctacatcaatctcaagg

a The negative symbols in the oligonucleotide names indicate the polarities of the nucleic acids to which the oligonucleotides anneal.
b Polarity of the oligonucleotide relative to the positive-strand viral genome.

Table S2. Oligonucleotides used for RT-qPCR

Oligonucleotides[a]	Polarity[b]	Sequence (5' to 3')
BCV 14427(-)	−	gctgctgatccagctttgcatgtagc
BCV 14588(+)	+	cagatcaactgaactaccctctttaagcagg
BCV 29397(-)	−	atgtcttttactcctggtaagcaa
BCV 29590(+)	+	gaaactgagtaattccagagaacc
H18s rRNA(-)	−	cattcgaacgtctgccctatc
H18s rRNA(+)	+	gcctgctgccttccttggatgtggtagcc

a The positive and negative symbols in the oligonucleotide names indicate the polarities of the nucleic acids to which the oligonucleotides anneal.
b Polarity of the oligonucleotide relative to the positive-strand viral genome.
